# Intraovarian Injection of Recombinant Human Follicle-Stimulating Hormone for Luteal-Phase Ovarian Stimulation during Oocyte Retrieval Is Effective in Women with Impending Ovarian Failure and Diminished Ovarian Reserve

**DOI:** 10.3390/biomedicines10061312

**Published:** 2022-06-03

**Authors:** Chao-Chin Hsu, Isabel Hsu, Li-Hsuan Lee, Yuan-Shuo Hsueh, Chih-Ying Lin, Hui Hua Chang

**Affiliations:** 1 Taiwan United Birth-Promoting Experts Fertility Clinic, Tainan 710, Taiwan; 2 Department of Obstetrics and Gynecology, National Taiwan University Hospital, Taipei 100, Taiwan; izserx@gmail.com; 3 Department of Obstetrics and Gynecology, National Cheng Kung University Hospital, Tainan 701, Taiwan; 4 Nepean Hospital, Penrith, NSW 2747, Australia; lihsuan.lee@health.nsw.gov.au; 5 Department of Medical Science Industries, College of Health Sciences, Chang Jung Christian University, Tainan 711, Taiwan; yshsueh@mail.cjcu.edu.tw; 6 Institute of Clinical Pharmacy and Pharmaceutical Sciences, College of Medicine, National Cheng Kung University, Tainan 701, Taiwan; ilg811280@gmail.com; 7 School of Pharmacy, College of Medicine, National Cheng Kung University, Tainan 701, Taiwan; 8 Department of Pharmacy, National Cheng Kung University Hospital, College of Medicine, National Cheng Kung University, Tainan 701, Taiwan; 9 Department of Pharmacy, National Cheng Kung University Hospital, Dou-Liou Branch, Yunlin 640, Taiwan

**Keywords:** diminished ovarian reserve, impending ovarian failure, intraovarian, luteal phase, recombinant human follicle-stimulating hormone, controlled ovulation stimulation, DuoStim

## Abstract

It is a challenge to obtain sufficient eggs during in vitro fertilization (IVF) in women with impending ovarian failure (IOF)/diminished ovarian reserve (DOR). Although studies have suggested that more than one wave of follicle growth exists, the efficacy of controlled ovulation stimulation (COS) in both follicular and luteal phases of the same ovarian cycle (DuoStim) is not established in women with IOF/DOR. We investigated the efficacy of DuoStim using the intraovarian injection of recombinant human follicle-stimulating hormone (rhFSH) during oocyte retrieval in women with DOR. For luteal-phase stimulation, intraovarian (Group A, N = 28) or superficial subcutaneous (Group B, N = 18) injection of 300 IU rhFSH immediately after oocyte retrieval was administered as the first dose, and intermittent superficial subcutaneous addition of gonadotropins was employed accordingly for further COS in both groups. In Group A, significantly lower Gn doses, a shorter duration of COS, a greater number of antral follicle counts, and an increased number of retrieved mature and total oocytes were noted. Compared with the clinical outcomes of luteal-phase COS, the average daily doses of rhFSH used in Group A were significantly lower. In summary, the novel approach using intraovarian rhFSH injection provides an efficient treatment regimen in women with IOF/DOR.

## 1. Introduction

Diminished ovarian reserve (DOR) is present in 10–40% of women with infertility, in which the ovary loses its normal reproductive potential, resulting in difficulty to achieve conception and menstrual cycle abnormalities [1]. In women with DOR, there is a reduced quantity and quality of oocytes produced by the ovaries, leading to poor-quality embryos [2]. Women with DOR are characterized with abnormal ovarian reserve tests presented by antral follicular count (AFC) < 5–7 follicles or anti-Mullerian hormone (AMH) < 0.5–1.1 ng/mL [3]. Various causative factors lead to DOR, and the most important factors are increasing age and ovarian surgeries, especially in women suffering from severe endometriosis [4]. The impending ovarian failure (IOF) or premature ovarian insufficiency (POI) represented the most severe forms of DOR. An early study indicated that an FSH value ≥ 10 IU/L on Day 3 of the menstrual cycle is prominent in women with imminent or IOF [5]. However, up to now, there is no definite consensus on terminology for women with very poor ovarian reserve, which includes an elevated serum FSH over 20 IU/L, a barely detectable AMH level (<0.02–0.2 ng/mL), and very low AFC (0–3 in number). In this study, we use “impending ovarian failure” to describe these women’s fertility status.

To obtain higher quality and quantity of oocytes and embryos in women with DOR, various treatment scenarios have been used [6,7], including high-dose recombinant human follicle-stimulating hormone (rhFSH) treatment, luteinizing hormone (LH) supplementation, the gonadotropin-releasing hormone (GnRH) antagonist cycle, and the use of adjuvant treatments, such as estrogen priming, growth hormone in controlled ovarian stimulation (COS) [6,7]. In 2003, based on ultrasonographic studies, two-to-three waves of follicular growth during the ovulatory period of healthy women has been noted [8]. The researchers suggested that follicles developing during the luteal phase may have the potential to ovulate in the presence of an LH surge. This fact brought new possibilities for controlled ovarian hyperstimulation especially for DOR women and for women requesting urgent fertility preservation prior to the oncology treatments. Clinical ovarian stimulation with human menopausal gonadotropin and letrozole during the luteal phase for women undergoing in vitro fertilization (IVF)/intracytoplasmic sperm injection (ICSI) treatment has been demonstrated to be effective for obtaining oocytes and, in turn, an increased number of embryos, from the second follicular wave for cryopreservation [9]. The in vitro developmental competence in the blastocyst stage was also similar to that of conventional COS [10]. Higher numbers of oocytes and good-quality embryos employing double stimulation (DuoStim) in a single menstrual cycle have recently been published in systematic reviews [11,12]. A recent multicenter study has confirmed that DuoStim protocol (luteal-phase COS after follicular-phase COS) derived oocytes are similar concerning developmental competence up to the blastocyst stage and is a feasible and efficient approach from clinical, obstetric, and perinatal perspectives. The DuoStim can be employed in women for emergent fertility preservation due to oncology treatment and in advanced maternal age and/or reduced ovarian reserve women who request urgent treatment in the shortest time frame [13]. The same study group further proved that DuoStim was more cost-effective than the conventional approach, with a higher cumulative live birth rate and a lower drop-out rate for further treatments in advanced maternal age/poor ovarian reserve women [13,14].

**Figure 1:**
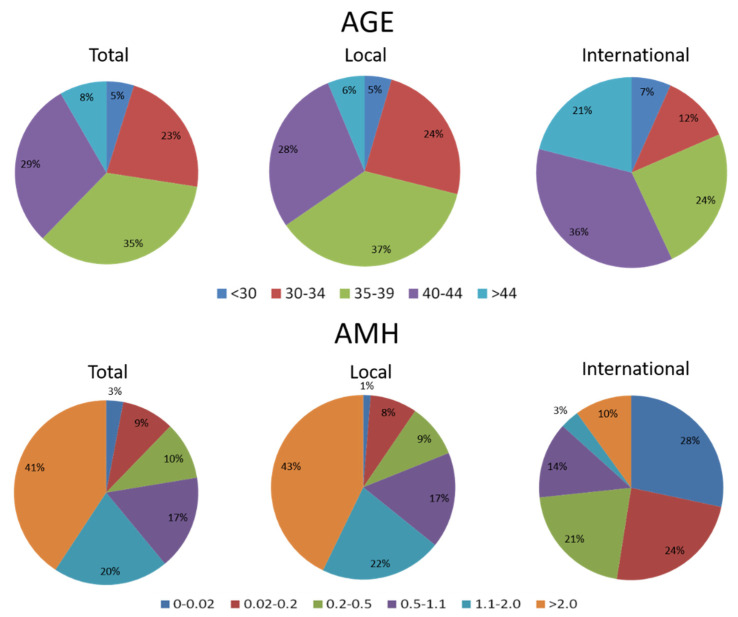
The distribution of age and anti-Mullerian hormone (AMH) levels among women who received assisted reproduction in our unit between 2015 and 2019. The percentage of advanced maternal age (≥35 years old) was 71% and 81% in local and international patients, respectively. The percentage of them with poor ovarian reserve is 87% in international patients (including 21% diminished ovarian reserve, and 52% impending ovarian failure with AMH ≤ 0.2). Local: Taiwanese patients, International: mostly Mainland China patients.

However, the efficacy of luteal-phase DuoStim in patients with DOR/IOF has not yet been examined. The enhancement of folliculogenesis has recently been shown in women with POI and early menopausal women who received intraovarian administration of rhFSH with platelet-rich plasma (PRP) [15,16]. We, therefore, hypothesized that more oocytes could be obtained by continuing ovarian stimulation after the first oocyte retrieval under direct ovarian administration of rhFSH. Our unit was the first assisted reproductive unit for international patients approved by the Ministry of Health and Welfare Taiwan Government to legally treat international patients in 2016. Since then, many advanced-aged couples, mostly from Mainland China, who presented with DOR/IOF, visited our unit for further conception after China ended the one-child policy in 2016 (Figure 1). Thus, we conducted a pilot study to investigate the efficacy of DuoStim both in the follicular and luteal phases in patients with DOR/IOF undergoing IVF treatments.

## 2. Materials and Methods

### 2.1. Ethics Approval

All procedures performed in this study are in accordance with the Declaration of Helsinki Good Clinical Practice. Local regulatory requirements were met and approved by the Institutional Review Board (TSMH IRB/Protocol No: 18-115-B). All patients were treated at TUBE Fertility Clinic, Tainan, Taiwan, under a license from the Ministry of Health and Welfare, Taiwan Government. All participants enrolled were consulted about the potential adverse reactions of the administration mode of gonadotropin (Gn) and signed a written consent form. Potential risks, including ovarian hyperstimulation (OHSS) and local reactions including skin irritation and ecchymosis, were fully explained.

### 2.2. Study Population and Design

#### 2.2.1. Participants

Women with DOR/IOF who requested IVF treatments from January 2017 to December 2019 in our unit were approached. The inclusion criteria were infertile women aged over 35 years, with a body mass index (BMI) of 17.0–28.0 kg/m^2^, AFC < 5 on Days 2–3 of the menstrual cycle, serum AMH concentration ≤ 0.5 ng/mL, and basal serum FSH concentration ≥ 10 IU/L. The exclusion criteria included women with an allergic history, coagulation disorders, and the use of hormonal preparations within the last 3 menstrual cycles.

#### 2.2.2. Controlled Ovarian Stimulation

**Figure 2:**
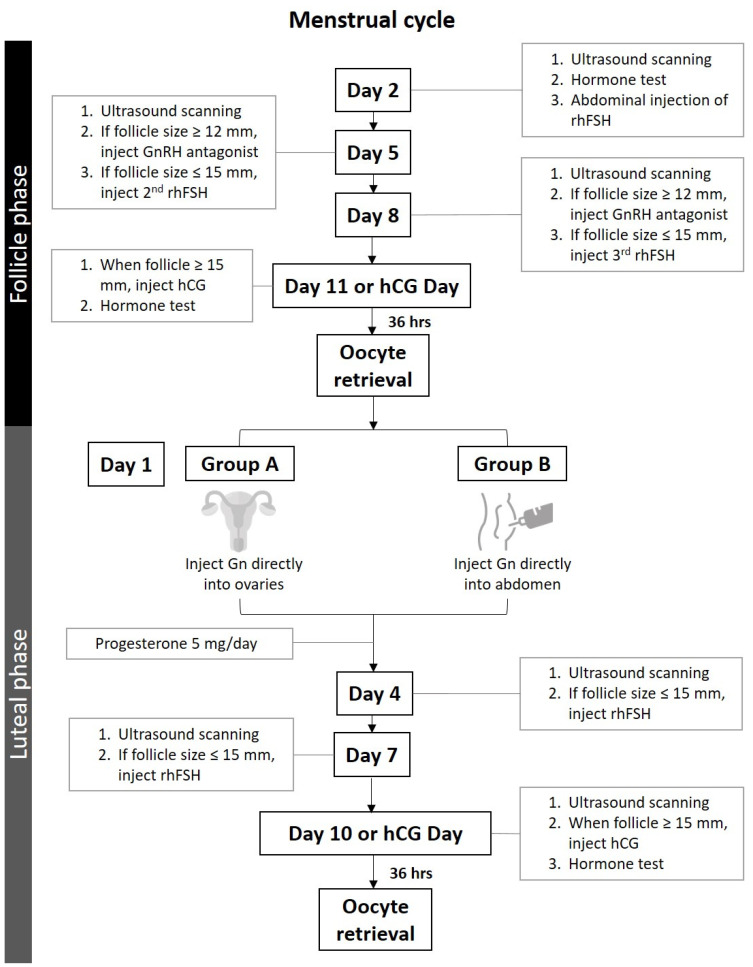
Overall scheme of the study design. Abbreviations: rhFSH: recombinant human follicle-stimulating; GnRH antagonist: gonadotropin-releasing hormone antagonist; hCG: human chorionic gonadotropin.

DuoStim-COS was employed in this study (Figure 2), and all enrolled subjects first received our routine COS starting in the early follicular phase using the gonadotropin-releasing hormone (GnRH) antagonist protocol. Double stimulation was employed immediately following the oocyte retrieval procedure in the operating room. The routine COS at our IVF unit is intermittent superficial subcutaneous/intradermal administration (the term intradermal administration will be used in the main article) of rhFSH [17]. The injection of Gn was performed on Days 2, 5, and 8 of the IVF cycle in accordance with our routine COS method [17]. In brief, 300 IU rhFSH (Gonal-f Prefilled Pen 300 IU rhFSH in 0.5 mL, Merck Serono S.p.A., Modugno, Italy) in combination with Menopur 375 IU (Menopur 75 IU, corresponding to FSH 75 IU and LH 75 IU; FerringGmBH, Kiel, Germany) reconstituted in 1 mL solvent was aspirated into a 2.5 mL syringe. The 30-gauge x 0.5-inch (0.31 × 13 mm) needle was tilted at 15° to deposit the Gn in the dermis or superficial subcutaneous layer at a depth of 1–2 mm under the skin of the lower abdominal wall. Women attended the clinic on Days 2, 5, 8, and 11 of their menstrual cycles for the administration of rhFSH and ultrasonography follow-up of ovarian follicle growth. Measurements of follicle diameter (mean diameter measured in two dimensions) under 5.0-megahertz transvaginal ultrasonography (Aloka 900, Tokyo, Japan) were performed by the same physician for follicular growth. On Days 5 and 8, if follicular growth did not meet the criteria (one follicle ≥ 15 mm) for egg retrieval, a second and third dose of Gn injection were given with the same administration mode, as previously described. The dosage for the second and third Gn injections was the same as that used for the first dose. Once the ovarian follicle reached 12 mm in size, the GnRH antagonist (orgalutron 0.25 mg, Vetter Pharma-Fertigung GmbH & Co. KG, Ravensburg, Germany) was initiated. Serum hormone profiles were obtained on Day 2 of the menstrual cycle and on the day of human chorionic gonadotropin (hCG) injection.

After oocyte retrieval of follicular-phase COS, participants were assigned to Groups A and B for their luteal-phase COS. The first dose of Gn (Gonal-F 300 IU) in Group A was injected directly into the ovaries immediately after oocyte retrieval. While in Group B, Gn was injected intradermally immediately after oocyte retrieval (Figure 2). The follow-up of the growth of follicles and administration of second or third doses of Gn employing intradermal Gn administration in both groups of women was in accordance with that performed in the follicular phase. Progesterone (medroxyprogesterone acetate 5 mg/day; Pfizer, Ascoli Piceno, Italy) was administered until the maturation of follicles in luteal-phase COS.

The oocyte retrieval procedures were carried out in accordance with our routine method [17]. In brief, oocyte retrieval was performed 36 h after triggering the final follicular maturation using 6500 IU recombinant hCG (Ovidriel, Merck SeronoS.p.A., Modugno, Italy) when at least one follicle reached ≥ 15 mm in diameter. Most of the mature oocytes obtained were cryopreserved. The immature oocytes were cultured for 24 h and then cryopreserved if mature. ICSI was used to fertilize a small portion of mature oocytes. The continuous single culture medium—(NX complete CSCM-NXC with gentamicin and HAS; FUJIFILM Irvine Scientific, Santa Ana, CA 92705, USA) was used for insemination and embryo culture. Fertilized pre-embryos were cultured to Day 3 cleavage-stage embryos and cryopreserved. The Kitazato Cryotech vitrification method was employed with the maximal of two oocytes or embryos per Cryotop (VT 601; KITAZATO Shizouka 416-0907, Japan). The safety endpoints including the proportion of women with moderate/severe-grade ovarian hyperstimulation syndrome (OHSS), and adverse events during the rhFSH treatments such as pain or skin reactions were recorded.

#### 2.2.3. Clinical Outcome

The primary outcomes include the total dosage of Gn used, the length of COS, serum FSH and estradiol (E2) levels, and targeted ovarian response (follicular growth and number of mature oocytes retrieved). The secondary outcomes are the total number of oocytes and embryos cryopreserved. The ovarian sensitivity index (OSI: the dose of rhFSH used divided by the number of mature oocytes obtained) [18], the follicular output rate (FORT: the preovulatory follicle count (14–22 mm in diameter) on hCG day × 100 divided by the small antral follicle count (3–8 mm in diameter) at baseline [19], and the follicle-to-oocyte index (FOI: the number of oocytes obtained divided by the number of antral follicles at the beginning of COS) [20] were used to represent the dynamic aspects of the follicular response to the DuoStim COS.

#### 2.2.4. Measurement of Serum Hormone Levels

A sequential immunoenzymatic two-step “sandwich” assay, the Beckman Coulter ACCESS immunoassay (UniCelDxl 800, Beckman Coulter, Brea, CA, USA), was used to measure FSH and luteinizing hormone (LH) serum levels. The lowest level of 0.2 IU/L could be detected with a ≤10% imprecision. On the other hand, a 1-step immunoenzymatic assay was applied to measure anti-Mullerian hormone (AMH) serum levels and can detect any levels ≤ 0.02 ng/mL with a ≤ 10.0% imprecision rate at concentrations ≥ 0.16 ng/mL. For estradiol and progesterone serum levels, a competitive binding immunoenzymatic assay was used for analysis with the lowest detectable levels of 20 pg/mL for estradiol and 0.10 ng/mL for progesterone.

### 2.3. Statistical Analysis

The Statistical Package for Social Sciences 23.0 (SPSS Inc., Chicago, IL, USA) was used. Categorical variables were expressed as numbers and percentages and assessed using chi-square tests. Continuous variables are expressed as the means ± standard deviations (SD) and assessed using *t*-tests or paired *t*-tests. The significant level was set at 0.05 for two-sided tests.

## 3. Results

### 3.1. Demographics of the Participants

In total, 921 women underwent IVF treatments between 2017 and 2019 at our unit; among them, 57 women who presented with DOR/IOF were approached. Among these women, 46 agreed to participate in the new treatment modes. In total, 28 women were in Group A (luteal-phase intraovarian injection of rhFSH for the first dose of DuoStim), and 18 women were in Group B (luteal-phase intradermal administration of rhFSH). The demographic characteristics of the subjects are shown in Table 1. The causative factors of infertility included ovulatory dysfunction in all participants, and other factors are listed in Table 2. Both groups of women had IOF with very low serum AMH levels (0.12 ± 0.11 and 0.13 ± 0.13 pg/mL) and elevated serum FSH levels (23.21 ± 11.73 and 23.41 ± 16.49 mIU/mL, respectively).

### 3.2. Clinical Response of Follicular-Phase COS in the Two Groups of Patients

Although slightly higher antral follicle counts in Group B, the total Gn doses used (2142.9 ± 690.5 vs. 1900.0 ± 896.3 IU in Groups A and B, respectively) and average daily doses of Gn required in both groups were comparable. However, a slightly shorter duration of follicular-phase COS (10.8 ± 3.4 vs. 8.8 ± 2.7 days, *p* = 0.044) was noted in Group B (Table 1). Follicle growth based on follicle size and serum estradiol levels was not different between the two groups. Significantly greater numbers of mature oocytes were retrieved in Group B (0.6 ± 0.8 vs. 1.2 ± 0.9, *p* = 0.017), but the number of oocytes and cryopreserved embryos were similar. In follicular-phase COS, premature ovulation was noted in 15 women in Group A and 3 in Group B. Empty follicles, defined as no oocytes obtained after extensive flushing during oocyte retrieval, were noted in 4 out of 28 women in Group A and in 1 out of 18 women in Group B.

### 3.3. Comparison of the Clinical Response between Intradermal Administration of rhFSH at the Follicular Phase and Intraovarian Administration of rhFSH as the First Dose at the Luteal Phase in Group A

Considerably lower rhFSH doses were used in the luteal phase in which intraovarian administration of rhFSH as the first dose of COS was performed (1100.9 ± 620.8 vs. 2142.9 ± 690.5 IU, *p* < 0.001), whereas the average daily doses of rhFSH required were similar. The duration of COS was significantly shorter in luteal-phase COS (6.2 ± 3.4 vs. 10.8 ± 3.5 days, *p* < 0.001) (Table 3).

In addition, the antral follicle counts were higher at the start of luteal-phase COS than the follicular-phase COS (2.6 ± 1.6 vs. 1.5 ± 1.3, *p* = 0.007). The growth of follicles based on the number of follicles ≥ 13 mm and maximal serum estradiol concentrations was similar in the follicular and luteal phases. The number of mature (1.6 ± 1.8 vs. 0.6 ± 0.8, *p* = 0.015) or total oocytes (2.1 ± 2.2 vs. 1.0 ± 0.9, *p* = 0.013) retrieved and total numbers of oocytes or embryos cryopreserved (1.5 ± 1.5 vs. 0.8 ± 0.9, *p* = 0.068) were higher in luteal-phase COS in which intraovarian administration of rhFSH as the first dose of COS was performed. Moreover, this effect was reflected by the considerably lower OSI and slightly higher FOI in luteal-phase COS (Table 3). The significantly higher serum levels of progesterone (P4) and lower levels of LH in luteal-phase COS were expected. Compared with premature ovulation in 15 women in follicular-phase COS, only 6 women experienced premature ovulation in their luteal-phase COS, adopting intraovarian administration of rhFSH as the first dose of COS. In addition, one woman ovulated during oocyte retrieval, one exhibited no growth of follicles, and another presented with empty follicles. No oocytes were obtained from these three women.

Through follicular-phase COS in Group A, cryopreservation of oocytes was performed in 14 women, and cryopreservation of embryos was performed in 4 women. Cryopreservation of oocytes was performed in 15 women, and cryopreservation of embryos was performed in 23 women in Group A in luteal-phase COS. Only eight participants asked for embryo transfer, among which three did not have blastocyst embryos; embryo transfer was not performed. Five women underwent embryo transfer, and one woman achieved clinical pregnancy, which unfortunately resulted in a miscarriage at a gestational age of 7 weeks.

Regarding adverse reactions, no discomfort, bleeding, or painful sensation was noted in women who received intraovarian administration of rhFSH. No women presented with symptoms or signs of OHSS in either follicular-phase or luteal-phase COS.

### 3.4. Comparison of the Clinical Response to Intradermal Administration of rhFSH between the Follicular Phase and Luteal Phase in Group B

The total rhFSH doses used were slightly lower in luteal-phase COS (1487.5 ± 695.6 vs. 1900.0 ± 896.3 IU, *p* = 0.067), whereas the average daily doses of rhFSH required were not different. The duration of COS was significantly shorter in luteal-phase COS (6.5 ± 3.1 vs. 8.6 ± 2.6 days, *p* = 0.017) (Table 3).

The antral follicle counts, growth of follicles based on the number of follicles ≥ 13 mm, and maximal serum estradiol concentrations were similar in follicular and luteal phases. In addition, although the number of mature oocytes (2.1 ± 2.5 vs. 1.2 ± 0.9) was similar, the total oocytes (3.0 ± 2.7 vs. 1.6 ± 1.2, *p* = 0.021) retrieved and the total numbers of oocytes and embryos cryopreserved (2.4 ± 2.4 vs. 1.1 ± 1.0, *p* = 0.024) were higher in luteal-phase COS. This effect was reflected by the lower OSI in luteal-phase COS (Table 3).

Compared with premature ovulation in three women in follicular-phase COS, no woman experienced premature ovulation in luteal-phase COS with intradermal administration of rhFSH. Similar to the findings in Group A, one woman ovulated during oocyte retrieval, one woman had no growth of follicles, and another presented with empty follicles. No oocytes were obtained from these three women in Group B. In Group B, follicular-phase COS, cryopreservation of oocytes was performed in nine women, and cryopreservation of embryos was performed in four women. In Group B, luteal-phase COS, cryopreservation of oocytes was performed in 11 women, and cryopreservation of embryos was performed in 4 women. Only one woman underwent embryo transfer in Group B, with failed results.

Regarding adverse reactions, no women presented with symptoms or signs of OHSS in either follicular-phase or luteal-phase COS.

### 3.5. Comparison of Clinical Response between Intraovarian and Intradermal Administration of rhFSH during Luteal-Phase COS

The clinical outcomes of luteal-phase COS were not significantly different between intraovarian and intradermal administration of rhFSH, except for the average daily doses of Gn required (Table 3). The average daily doses of Gn required in Group A were significantly lower than those used in Group B (192.1 ± 72.4 IU vs. 246.7 ± 97.3 IU, *p* = 0.043). The total dose of rhFSH used was marginally significant (*p* = 0.059). Significant differences regarding the total dose of Gn (714.4 ± 436.0 IU vs. 1487.5 ± 695.6 IU, *p* < 0.001), and OSI (331.91 ± 246.15 IU vs. 580.63 ± 314.82 IU, *p* = 0.008) were noted after retrograde adjustment of the parameters with follicular-phase antral follicle numbers (Table 3).

The efficacy of luteal-phase COS was noted by the OSI of 541.25 ± 287.17 and 580.63 ± 314.82 IU in Groups A and B, respectively, in comparison with those in follicular-phase COS of 1610.00 ± 1011.36 and 1179.33 ± 789.91 IU in Groups A and B, respectively. We also observed a FORT value of 56–73% and FOI of approximately 84–93% in the luteal-phase COS, indicating the dynamic response of ovarian folliculogenesis to exogenous Gn and the efficacy of both administration modes.

## 4. Discussion

In this study, the novel application of intraovarian administration of rhFSH was efficiently employed in luteal-phase COS in women with DOR/IOF. More oocytes were obtained, and more embryos were cryopreserved for future embryo transfer. The results also suggested advantages for women with DOR/IOF receiving an intraovarian injection of rhFSH in the luteal phase, including (1) direct administration of rhFSH to the target organ, the ovary itself; (2) the lack of discomfort as rhFSH injection was performed under intravenous sedation and all patients were unaware of the processes; (3) a friendlier approach with the avoidance of multiple daily injections of Gn, compared with that used in conventional COS; (4) a relatively short COS period, less than 7 days; and (5) a lower dose of Gn used. In conclusion, our findings show that intraovarian administration of rhFSH as the first dose in luteal-phase COS may provide an alternative treatment regimen in women.

The “random-start” protocol, i.e., starting COS at any time in the menstrual cycle, was developed for urgent fertility preservation before starting oncology treatments [21]. The number of oocytes obtained from the “random-start” protocol was equal to that of COS started in the early follicular phase [22]. In women with low oocyte yield, DuoStim was recommended for the purpose of oocyte or embryo accumulation, especially in women >40 years old [10,20,23]. One study also showed that almost doubled numbers of oocytes retrieved (3.5 vs. 1.7 in luteal and follicular phases, respectively) employing DuoStim in the same menstrual cycle, which provided more opportunities for accumulating oocytes in poor responders [24]. According to the Bologna criteria, the poor ovarian responders (POR) represent a subgroup of women with relatively poor pregnancy prognosis and only a 6.8–7.9% of live birth rate in the IVF cycle [7,25]. In the present study, we studied another group of women with DOR/IOF whose serum AMH levels were minimally detectable, at an average of 0.12 ng/mL. Results indicated that intraovarian administration of rhFSH as the first dose in luteal-phase COS worked efficiently, with more cryopreserved oocytes and embryos for future use.

Regarding the regimen of DuoStim, most of the previous studies have reported an average of 3–5-day break from the day of oocyte retrieval to the start of luteal COS [24]. In our study, the luteal-phase ovarian stimulation was immediately initiated after the first oocyte retrieval and was not only well-tolerated but also provided even more oocytes and embryos than those obtained in the follicular-phase COS. Therefore, intraovarian administration of rhFSH in luteal-phase COS is an alternative option for women who have undergone recurrent failed oocyte retrieval procedures or for women with insufficient ovarian responses using conventional IVF protocols. Regarding the dose of Gn, a previous study reported that approximately two times the dose of Gn was required per oocyte retrieved in luteal-phase COS in comparison to that of follicular-phase COS [24,26]. One study has also indicated that the surge of FSH and LH in the pituitary gland induced by the same dose of GnRH agonist was much higher in the first trigger than in the second trigger [27]. Thus, reduced ovarian sensitivity to Gn stimulation during luteal-phase ovarian stimulation was noted [24]. However, a much lower dose of Gn was required to complete luteal-phase COS employing intraovarian administration of rhFSH in this study. It is possible that intraovarian administration of rhFSH can result in more efficient binding of the FSH receptor (FSHR) and yield greater stimulating effects on the growth of ovarian follicles. Although the safety of DuoStim was a concern [28], our results have shown that intraovarian administration of rhFSH in luteal-phase COS worked efficiently and safely in women with DOR/IOF. Taken together, luteal-phase COS employing this intraovarian protocol for DuoStim provides an efficient option to accumulate much more oocytes in women with DOR/IOF.

In the era of increasing demand for more women, many with advanced age, with DOR/IOF request IVF treatments, different administration modes of rhFSH might optimize their response to COS. Our recent studies by administrating rhFSH in combination with PRP directly into the target ovarian tissue and also whole dimension subcortical ovarian administration indicated the restoration of ovarian function and clinical pregnancy in women with premature ovarian insufficiency and early menopause [15,16]. We have also demonstrated that both intradermally and intravaginally administered rhFSH can be absorbed efficiently and enhance ovarian follicle growth in POR women [17,19,29,30,31]. Thus, a personalized IVF treatment with fine-tuning of the Gn dose in accordance with each individual’s ovarian response has become a safe and effective practice [32,33]. In this study, reducing the total dose of Gn administered by employing intraovarian injection greatly reduces the cost of each cycle. Furthermore, the decrease in the number of injections needed for COS could greatly reduce the emotional stress experienced by infertile women [34,35]. 

It has been shown that FSH treatment results in the proliferation of ovarian surface epithelium (OSE) via FSHR on OSE cells [36,37]. The binding of FSH and FSHR could lead to enhanced gene expression profiles and induce growth promotion in OSE cells [37]. An increased number of stem cells in combination with increased proliferation of OSE after FSH treatment was noted in tissue samples from a 60-year-old woman. That study indicated that once the inhibitory factors in situ were overcome, the in vitro cultured stem cells from aged ovaries were shown to have the ability to differentiate into oocyte-like structures [38]. The presence of stem cells in the OSE of postmenopausal women was also noted in another study, in which stem cells in the OSE differentiate into oocytes and parthenote embryos in culture [39,40]. Studies also indicated that FSH exerts direct action through FSHR3 expressed on OSE cells through the MAPK–ERK pathway, in addition to a more popular mechanism via the cAMP pathway through FSHR1 expressed on granulosa cells of growing follicles [41]. A recent study indicated that the response of granulosa cells to FSH involves a complex and coordinated program of hundreds of genes [42,43]. Many signaling pathways were found regarding Gn and follicle growth in the ovary [41]. It has been suggested that the microenvironment, which provides the required growth factors/cytokines supporting the stem cells is compromised with age, and stem cells are not able to differentiate into oocytes [44,45]. The stem cells from aged ovaries have been demonstrated to retain differentiation potential and form oocytes when exposed to a young ovarian environment [46]. Whether the direct injection of rhFSH in the present study modulates gene expression, transcription factors, and signaling pathways for proper folliculogenesis requires future investigation.

The present study has certain limitations, including a relatively small sample size and a short follow-up period. Most of the retrieved oocytes were cryopreserved and stored for further in vitro fertilization and embryo transfer. However, their treatments were put on hold due to the outbreak of COVID-19. In addition, given that we did not regularly use the conventional daily injection of Gn for COS, a comparison of the efficacy of this novel intraovarian administration of Gn and conventional daily injections during the luteal phase could not be performed. There were also aspects that should be assessed in future studies including (1) identification of the most effective dose for intraovarian administration of rhFSH; (2) assessment of a selection of the optimal interval for the successive second and third doses of rhFSH/Gn; (3) assessment of whether the addition of growth hormone, clomiphene citrate or letrozole in combination with intraovarian administration of rhFSH is helpful and more effective for women with DOR/IOF; (4) determination of whether the effectiveness of intraovarian administration of rhFSH requires an observation period of a few months given that 5–6 months were required for primordial growth to early antral follicles based on the mechanism of folliculogenesis; and (5) determination of whether intraovarian administration of rhFSH is also effective in the follicular-phase or random-start COS. Thus, further prospective studies are needed to clarify these issues in individual patients with DOR/IOF. Regarding the safety of Gn, no teratogenic, mutagenic, or clastogenic effects were noted for women undergoing IVF from a series of trials and meta-analyses [47,48,49]. As a high concentration of Gn/rhFSH could even lead to adverse/inhibitory effects on follicular growth [50], the dose of Gn to be injected directly into ovarian tissue should be carefully monitored. However, direct administration of Gn to its target organ—the ovary—has not been carried out in other previous studies. Thus, future well-designed prospective studies should be conducted to illustrate the safety and effectiveness of this novel administration mode.

## 5. Conclusions

Our results provide a useful model of intraovarian administration of rhFSH efficiently employed in luteal-phase COS in women with DOR/IOF. The data also demonstrated the feasibility of initiating ovarian stimulation immediately after oocyte retrieval with rhFSH to obtain more oocytes and embryos in women with DOR/IOF. The new approach is also promising for patients undergoing emergent fertility preservation.

## Figures and Tables

**Table 1:** The demographic characteristics and clinical response of the subjects.

Characteristics	Group A (*n* = 28)	Group B (*n* = 18)	t	*p* Value
	Mean ± SD	Mean ± SD		
Age (y/o)	42.72 ± 3.83	42.17 ± 4.64	0.447	0.657
BMI (kg/m^2^)	22.12 ± 2.62	22.24 ± 3.48	−0.126	0.901
AMH (ng/mL)	0.12 ± 0.11	0.13 ± 0.13	−0.367	0.716
FSH_day 2 (IU/L)	23.21 ± 11.73	23.41 ± 16.49	−0.049	0.961
F_AFC(number)	1.5 ± 1.3	2.4 ± 1.5	−2.266	0.028 *
F_total dose of Gn (IU)	2142.9 ± 690.5	1900.0 ± 896.3	1.035	0.306
Follicular-phase days	10.8 ± 3.4	8.8 ± 2.7	2.073	0.044 *
Daily doses of Gn ^‡^ (IU/days)	204.3 ± 45.7	214.2 ± 65.8	−0.602	0.551
F_E2_day 2 (pg/mL)	35.6 ± 20.4	58.3 ± 34.9	−2.565	0.014 *
F_LH_ day 2 (IU/L)	6.07 ± 3.58	4.82 ± 3.47	1.103	0.277
F_P4_ day 2 (ng/mL)	0.47 ± 0.34	0.52 ± 0.37	−0.430	0.670
F_FSH_ day 2 (IU/L)	18.04 ± 8.01	17.10 ± 15.52	0.252	0.802
F_E2_hCGday (pg/mL)	387.3 ± 250.4	371.0 ± 296.6	0.111	0.912
F_LH_ hCGday (IU/L)	17.53 ± 29.89	11.16 ± 9.55	1.163	0.252
F_P4_ hCGday (ng/mL)	1.09 ± 1.96	0.85 ± 0.97	0.306	0.761
F_ Follicles < 11 mm (number)	0.7 ± 0.9	0.6 ± 0.9	0.291	0.773
F_ Follicles 12–15 mm (number)	0.5 ± 0.6	0.9 ± 1.3	−1.330	0.190
F_Follicles ≥ 16 mm (number)	1.0 ± 0.7	0.8 ± 0.7	0.893	0.376
F_ Mature oocytes (number)	0.6 ± 0.8	1.2 ± 0.9	−2.492	0.017 *
F_ Immature oocytes (number)	0.4 ± 0.7	0.4 ± 0.6	0.010	0.992
F_ Total oocytes (number)	1.0 ± 0.9	1.6 ± 1.2	−2.014	0.050
F_Eggs frozen (number)	0.6 ± 0.8	0.8 ± 1.0	−0.939	0.353
F_Embryos frozen (number)	0.2 ± 0.6	0.2 ± 0.4	0.197	0.845
F_Total frozen (number)	0.8 ± 0.9	1.1 ± 1.0	−0.848	0.401
OSI	1516.67 ± 653.86	1675.00 ± 1166.78	−0.616	0.544
OSI ^#^	1610.00 ± 1011.36	1179.33 ± 789.91	1.244	0.223
FORT	93.84 ± 42.21	82.38 ± 50.51	1.262	0.215
FOI	0.72 ± 0.78	0.75 ± 0.56	−0.089	0.929

Abbreviations: BMI: body mass index; AMH: anti-Mullerian hormone; FSH: follicle-stimulating hormone; F: follicular phase; AFC: antral follicular count; Gn: gonadotropin; E2: estradiol; P4: progesterone; LH: luteinizing hormone; hCG: human chorionic gonadotropin; OSI: ovarian sensitivity index, the dose of rhFSH used divided by the number of mature oocytes obtained; OSI ^#^: ovarian sensitivity index, the dose of rhFSH used divided by the number of total oocytes obtained; FORT: follicular output rate, the ratio of preovulatory follicle count (≥ 13 mm in diameter) on hCG day × 100/small antral follicle count (3–8 mm in diameter) at baseline; FOI: follicle-to-oocyte index, the number of oocytes obtained divided by the number of antral follicles at the beginning of COS. ^‡^ Total Gn dose divided by follicular-phase days; * *p* < 0.05.

**Table 2:** Other causative factors of infertility in our subjects besides ovarian dysfunction.

	Group A	Group B
	Number (*n* = 28)	Number (*n* = 18)
Endometriosis with chocolate cysts	5	2
Adenomyoma	5	2
Myoma	8	5
Teratoma	1	1
Hyperthyroidism	1	0
Hypothyroidism	0	1
Ovarian cyst	1	0
Endometrial polyp	1	2
Cervix dysplasia	0	1
Unexplained infertility	0	0

**Table 3:** Comparison of clinical response between the follicular phase and luteal phase in Group A and Group B.

	Group A (*n* = 28)				Group B (*n* = 18)				Comparison of Luteal-Phase COS ^3^	
	Follicular Phase/Intradermal Administration	Luteal Phase/Intraovarian Administration	t ^1^	*p* Value ^1^	Follicular Phase/Intradermal Administration	Luteal Phase/Intradermal Administration	t ^2^	*p* Value ^2^	t ^3^	*p* Value ^3^
Characteristics	Mean ± SD	Mean ± SD			Mean ± SD	Mean ± SD				
AFC (number)	1.5 ± 1.3	2.6 ± 1.6	−2.936	0.007 *	2.4 ± 1.5	3.1 ± 1.8	−1.394	0.181	−0.998	0.323
Total Gn dose(IU)	2142.9 ± 690.5	1100.9 ± 620.8	6.300	<0.001 *	1900.0 ± 896.3	1487.5 ± 695.6	1.947	0.067	−1.939	0.059
Luteal phase Total Gn dose ^5^ (IU)	-	714.4 ± 436.0	-	-	-	1487.5 ± 695.6	-	-	4.783	<0.001 *
Interval of egg retrieval (Follicular-phase days vs. Luteal-phase days)	10.8 ± 3.5	6.2 ± 3.4	5.450	<0.001 *	8.6 ± 2.6	6.5 ± 3.1	2.649	0.017 *	−0.219	0.828
Average daily doses of Gn required ^4^ (IU/days)	207.2 ± 43.9	192.1 ± 72.4	1.020	0.317	216.1 ± 67.3	246.7 ± 97.3	−1.173	0.258	−2.031	0.043 *
Average daily doses of Gn required ^5^ (IU/days)	-	119.4 ± 12.1	-	-	-	246.7 ± 97.3	-	-	6.263	<0.001 *
E2_hCG day (pg/mL)	387.3 ± 250.4	521.0 ± 447.4	−1.728	0.097	371.0 ± 296.6	565.1 ± 430.0	−1.887	0.077	−0.319	0.752
LH_ hCG day (IU/L)	17.53 ± 29.89	3.55 ± 3.29	2.098	0.050	11.16 ± 9.55	6.88 ± 8.02	1.368	0.194	−1.325	0.202
P4_ hCG day (ng/mL)	1.09 ± 1.96	12.36 ± 9.59	−5.903	<0.001 *	0.85 ± 0.97	4.55 ± 4.53	−3.137	0.009 *	1.701	0.099
hCG_Follicles ≥ 13 mm (number)	1.4 ± 0.6	1.6 ± 1.4	−0.613	0.545	1.8 ± 1.2	1.9 ± 1.7	−0.417	0.682	−0.829	0.412
Mature oocytes (number)	0.6 ± 0.8	1.6 ± 1.8	−2.601	0.015 *	1.2 ± 0.9	2.1 ± 2.5	−1.571	0.136	−0.624	0.536
Immature oocytes (number)	0.4 ± 0.7	0.5 ± 0.7	−0.722	0.477	0.4 ± 0.6	0.9 ± 1.1	−2.167	0.046 *	−1.287	0.209
Total oocytes (number)	1.0 ± 0.9	2.1 ± 2.2	−2.651	0.013 *	1.6 ± 1.2	3.0 ± 2.7	−2.569	0.021 *	−1.080	0.286
Frozen eggs (number)	0.6 ± 0.8	1.0 ± 1.5	−1.044	0.305	0.8 ± 1.0	1.9 ± 2.3	−2.361	0.030 *	−1.189	0.245
Frozen embryos (number)	0.2 ± 0.6	0.5 ± 1.0	−1.491	0.147	0.2 ± 0.4	0.6 ± 1.7	−0.941	0.361	−0.190	0.850
Total frozen (number)	0.8 ± 0.9	1.5 ± 1.5	−1.898	0.068	1.1 ± 1.0	2.4 ± 2.4	−2.489	0.024 *	−1.367	0.179
OSI	1516.67 ± 653.86	738.89 ± 331.22	3.092	0.015 *	1675.00 ± 1166.78	810.51 ± 437.79	2.359	0.040 *	−0.922	0.364
OSI ^#^	1610.00 ± 1011.36	541.25 ± 287.17	4.260	0.001 *	1179.33 ± 789.91	580.63 ± 314.82	2.474	0.029 *	0.348	0.730
Lut_OSI ^#,^^5^	-	331.91 ± 246.15	-	-	-	580.63 ± 314.82	-	-	2.761	0.008 *
FORT	93.84 ± 42.21	73.48 ± 47.21	1.702	0.103	82.38 ± 50.51	56.29 ± 40.87	2.011	0.066	0.693	0.492
FOI	0.72 ± 0.78	0.84 ± 0.78	−0.508	0.616	0.75 ± 0.56	0.93 ± 0.40	−1.138	0.274	−0.018	0.986

Abbreviations: AFC: antral follicular count; Gn: gonadotropin; E2: estradiol; P4: progesterone; LH: luteinizing hormone; hCG: human chorionic gonadotropin; OSI: ovarian sensitivity index, the dose of rhFSH used divided by the number of mature oocytes obtained; OSI ^#^: ovarian sensitivity index, the dose of rhFSH used divided by the number of total oocytes obtained; FORT: follicular output rate, the ratio of preovulatory follicle count (≥ 13 mm in diameter) on hCG day × 100/small antral follicle count (3–8 mm in diameter) at baseline. ^1^ The comparison of clinical response between follicular-phase intradermal administration of rhFSH and luteal-phase intraovarian administration of rhFSH in Group A. ^2^ Comparison of the clinical response between the follicular phase and luteal phase employing intradermal administration of rhFSH in Group B. ^3^ Comparison of the clinical response to intraovarian (Group A) or intradermal administration (Group B) of rhFSH in luteal-phase COS. ^4^ Total Gn dose divided by time interval of egg retrieval. ^5^ Total Gn dose divided by time interval of egg retrieval and adjusted by follicular-phase AFC: based on the concept that a lower Gn dose is required for those who have better ovarian reserve. Thus, total Gn used in the luteal phase of Group A was adjusted with initial follicular-phase AFC. Total Gn dose dividend by Group B AFC and then multiplied by Group A AFC. * *p* < 0.05.

## Data Availability

The data that support the findings of this study are available from Chao Chin Hsu. Restrictions apply to the availability of these data, which were used under license for this study. The data presented in this study are available on request from Chao Chin Hsu with the permission of the Taiwan United Birth-promoting Experts Fertility Clinic.
